# Good Psychometric Properties of the Addiction Version of the Revised Illness Perception Questionnaire for Health Care Professionals

**DOI:** 10.1371/journal.pone.0164262

**Published:** 2016-11-08

**Authors:** Astri Parawita Ayu, Boukje Dijkstra, Milou Golbach, Cor De Jong, Arnt Schellekens

**Affiliations:** 1 Department of Psychiatry, School of Medicine, Atma Jaya Catholic University of Indonesia, Jakarta, Indonesia; 2 Nijmegen Institute for Scientist-Pracititoners in Addiction, Radboud University, Nijmegen, The Netherlands; 3 Praktikon, Nijmegen, the Netherlands; 4 Department of Psychiatry, Radboud University Medical Centre, Nijmegen, the Netherlands; Medizinische Universitat Wien, AUSTRIA

## Abstract

**Background:**

Addiction, or substance dependence, is nowadays considered a chronic relapsing condition. However, perceptions of addiction vary widely, also among healthcare professionals. Perceptions of addiction are thought to contribute to attitude and stigma towards patients with addiction. However, studies into perceptions of addiction among healthcare professionals are limited and instruments for reliable assessment of their perceptions are lacking. The Illness Perception Questionnaire (IPQ) is widely used to evaluate perceptions of illness. The aim of this study was to evaluate the psychometric properties of the IPQ: factor structure, internal consistency, and discriminant validity, when applied to evaluate healthcare professionals’ perceptions of addiction.

**Methods:**

Participants were 1072 healthcare professionals in training and master students from the Netherlands and Indonesia, recruited from various addiction-training programs. The revised version of the IPQ was adapted to measure perceptions of addiction (IPQ-A). Maximum likelihood method was used to explore the best-fit IPQ factor structure. Internal consistency was evaluated for the final factors. The final factor structure was used to assess discriminant validity of the IPQ, by comparing illness perceptions of addiction between 1) medical students from the Netherlands and Indonesia, 2) medical students psychology students and educational science students from the Netherlands, and 3) participants with different training levels: medical students versus medical doctors.

**Results:**

Factor analysis revealed an eight-factor structure for the perception subscale (demoralization, timeline chronic, consequences, personal control, treatment control, illness coherence, timeline cyclical emotional representations) and a four-factor structure for the attribution subscale (psychological attributions, risk factors, smoking/alcohol, overwork). Internal reliability was acceptable to good. The IPQ-A was able to detect differences in perceptions between healthcare professionals from different cultural and educational background and level of training.

**Conclusions:**

The IPQ-A is a valid and reliable instrument to assess healthcare professionals’ perceptions of addiction.

## Introduction

Addiction, or substance dependence, is often considered a chronic relapsing condition, characterized by compulsive drug seeking and drug use, despite the many negative consequences it has for the individual (e.g. physical and mental wellbeing and social functioning) [1]. Addiction is a major global health problem with one year prevalence rates ranging between 3–7% [2]. It is often complicated by both physical and mental co-morbidities, including blood-borne infections, cardiovascular incidents, carcinoma, mood and anxiety disorders, psychosis, and personality disorders [3, 4]. Consequently, all kinds of healthcare professionals encounter patients with addiction.

Patients with addiction often receive negative attitudes and stigma [5]. Though experiences of rejection of patients with addiction were lowest for health care professionals, negative attitudes among health care professionals are more common towards patients with addiction, compared to other psychiatric and physical conditions [6, 7]. Moreover, these negative attitudes have been suggested to affect good quality of care for these patients [8, 9].

It has been suggested that perceptions about addiction among health professionals affect their attitudes and stigmatizing behavior towards these patients [10]. For example, the belief among healthcare professionals that a patient can control injecting drug use was associated with more negative attitudes towards such patients [10]. Studies indicate that perceptions of addiction vary widely among health professionals [11–13]. For example, some consider addiction as a chronic brain disease, resulting from genetic vulnerabilities and a change in brain function as a consequence of drug use [14, 15]. Others view addiction as a social problem, rather than an individual medical problem [16]. Others have a more moral viewpoint on addiction as a blameworthy condition, resulting from immoral behavior [17]. These examples of how health care professionals can perceive addiction are thought to relate to their attitudes towards patients with an addiction [10].

While several instruments are available for the assessment of attitudes and stigma, instruments for the assessment of illness perception among health care professionals are currently lacking. Yet, in the context of addiction it is of special interest to explore the role of perception in attitude and stigma, given the high variation in these perceptions. A better insight into perceptions of addiction among healthcare professionals and the relation with (stigmatizing) attitudes could help healthcare professionals to reflect on their perceptions and attitudes, and develop more professional attitudes towards these patients. As such, reliable assessment of perceptions of addiction among healthcare professionals could inform training programs for healthcare professionals eventually contributing to better care for patients with addiction.

Several instruments are available to assess individual illness perceptions, mainly applied in patients suffering various conditions [18–25]. The most commonly used instrument is the Illness Perception Questionnaire (IPQ), especially the revised version (IPQ-R) [26, 27]. For instance, the IPQ-R has been used to evaluate illness perceptions among patients suffering cancer [28], inflammatory bowel disease [29], epilepsy [30], mental disorder [31], and substance use disorders [32]. The IPQ-R has sparsely been applied to evaluate health professionals’ perceptions of schizophrenia [33].

The IPQ-R is based on the theory of self-regulation of Howard Leventhal [34]. This theoretical framework evaluates individual illness representations on five dimensions, represented by an identity subscale, seven perception subscales and an attribution subscale in the IPQ-R: 1) symptoms related to the illness (identity subscale), 2) the course of the illness (subscales timeline chronic, and cyclical), 3) cure and control of the illness (subscales patient and treatment control), 4) consequences of the illness for daily life and social function (subscales emotional representation, severe consequences), and 5) illness coherence, representing the understanding of the illness of the subject [18, 34, 35]. The attribution subscale identifies four domains of causality factors: psychological attribution, risk factors, immunity, and accident or chance.

Studies in various illnesses showed good psychometric properties of the IPQ-R. For example in survivors of esophageal cancer and people with a genetic predisposition to cancer the IPQ-R showed a good-fit with the factor structure outlined above [36] [37]. Similar findings were observed for general mental health problems (without specific diagnosis) [38] and specific mental disorders, such as schizophrenia [39, 40] and post-partum depression [41]. However, in Chinese injecting drug users the IPQ-R was less reliable, with poor-fit factor structure [32]. The one study applying the IPQ to study healthcare professionals’ illness perceptions studied schizophrenia showing a poor-fit factor structure and low internal reliability [33].

The aim of this study was to evaluate the psychometric properties of the IPQ-R, when applied to evaluate healthcare professionals’ perceptions of substance addiction. Specifically, the factor structure and reliability (internal consistency of factors) were investigated using a version of the IPQ-R adapted for addiction (IPQ-A), among a variety of healthcare professionals. Furthermore, the discriminant validity of the IPQ-A was investigated by comparing perceptions of addiction between healthcare professionals of different professional and cultural background and with different levels of training.

## Methods

### Participants

The current study involved 1072 participants from Indonesia and the Netherlands (mean age 24.7; 74.3% females). Participants were healthcare professionals and master students with different educational background (medical doctors, psychologists, nurses, and social workers; medicine, psychology, educational science respectively). They were recruited from various addiction-training programs in the Netherlands and Indonesia.

Participants from the Netherlands were from the master in addiction medicine training (*n* = 50), a conference for the occupational medicine specialists (*n* = 47), and an addiction workshop at a national addiction conference for psychology students, psychologists, nurses and social workers (*n* = 26). Furthermore, we delivered the questionnaire to general practitioner trainees (*n* = 68), clinical psychologist trainees (*n* = 47), and students of Radboud University: medicine (*n* = 107), psychology (*n* = 286), and educational science (*n* = 121). They were recruited during addiction lectures. All the participants were asked to fill in the questionnaire before the training. We delivered the questionnaire also to medical students in Indonesia. Third-year medical students were recruited from University of Padjajaran (*n* = 192) and Atma Jaya Catholic University of Indonesia (*n* = 128). They were asked to fill in the questionnaire in a classroom. Table 1 describes the characteristic of the respondents.

**Table 1:** Characteristics of the Respondents.

	Master in addiction medicine training	General practitioners training	Occupational medicine	Addiction Workshop (nurse, social worker, psychologist, psychology student)	Mental health psychology training	Student education science	Student psychology	Student medicine (Netherlands)	Student medicine (Indonesia)
Gender(*n*, %)									
Male	24(48)	16(23.5)	27(57.4)	6(23.1)	5(10.6)	4(3.3)	53(18.5)	29(27.1)	112(35)
Female	26(52)	52(76.5)	20(42.6)	20(76.9)	42(89.4)	117(96.7)	233(81.5)	78(72.9)	208(65)
Age,mean(SD)	41.3(9.73)	28.7(2.08)	51.5(8.34)	36.0(7.72)	29.7(6.06)	20.8(1.63)	21.8(1.78)	21.7(1.54)	20.8(1.04)
Response rate	100%	-	-	-	-	80.7%	96.5%	71,3%^a^	70.7%^a^

^a^estimated response rate, based on university data on total number of students

### Instrument

#### The Addiction version of Illness Perception Questionnaire (IPQ-A)

The IPQ-A is adapted from IPQ-R to evaluate healthcare professionals’ perceptions of substance addiction. The identity scale that assesses symptoms of illness experienced by the patient was excluded. The IPQ-A consists of two sections that assess domains of illness perception: perceptions and attributions [21]. We changed the title and the instruction in all sections substituting ‘illness’ for ‘substance addiction’. The first section of IPQ-A has 38 items about perceptions and the second section has 18 items about attributions. Response categories for all items ranged from 1 (strongly disagree) to 5 (strongly agree), as in the IPQ-R.

The content of items are the same as the IPQ-R but the structure was changed to be more appropriate for evaluating healthcare professionals’ perception. For example: ‘*The course of my illness depends on me’* became *‘The course of this illness depends on the patient’*. The third section contains the same list of 18 items as potential causes of addiction as the original attribution items in the IPQ-R. Examples are ‘stress or worry’, ‘bad luck’ and ‘alcohol use’. One of the authors (CDJ) adapted the IPQ-R into IPQ-A by making these changes and another author (AS) checked the adaptation. Both authors had a meeting to produce consensus on the final draft of the IPQ-A.

### Procedure

The final English version of the IPQ-A was translated into Indonesian and Dutch language following the World Health Organization guidelines for adaptation and translation of an instrument [42]. Two healthcare professional experts in addiction field in the Netherlands translated the English version into Dutch. They discussed their translation draft and produced one consensus Dutch version draft. An expert panel in addiction and psychometric study discussed the consensus draft and produced the final version. Then an independent translator translated it back into English, in order to check discrepancies. The same procedure was done to translate the IPQ-A into Indonesian language.

For the psychometric evaluation, we delivered the final version of the Indonesian and Dutch IPQ-A to healthcare professionals in training and students in each country. The participants filled in the IPQ-A in a room during their training session (for healthcare professionals) or in the classroom of their university (for students), after they received information about this study. The ethics committee faculty of social sciences Radboud University approved this study (ECSW2015-2508-333).

### Analyses

The maximum likelihood method with varimax rotation was performed to explore the IPQ-A factor structure, using all 38 items for perceptions and 18 items for attributions. Before running the maximum likelihood for the perception scale several items need to be reversed in line with the original IPQ-R [21]. The maximum likelihood was performed using all data of all groups together. The Chronbach’s alpha (internal consistency) was measured to evaluate the reliability of each factor. Chronbach’s alpha ≥.5 was considered acceptable, in line with previous studies [43, 44]. The correlations between the IPQ-A subscales were evaluated by calculating the Pearson’s correlation coefficient.

In order to test discriminant validity of the IPQ-A subscale scores were calculated using the new factor structure of the IPQ-A. Multivariate analysis of variance (MANOVA) was used to explore differences in illness perceptions between: 1) medical students from the Netherlands and Indonesia, 2) Dutch master students from different educational backgrounds (medicine, psychology, and educational science), and 3) medical students and medical doctors. Age and gender were used as covariates in the MANOVA analyses.

## Results

### Factor Structure and Internal Consistency

The maximum likelihood analysis identified eight factors for illness perception with eigenvalues greater than 1. Of the 38 items, one item (*the illness will improve in time*) was removed because it did not have a standardized factor loading above .40 on any factor. After deleting this item, the eight factors explained for a total of 58.31% of the items’ variance. Seven of the eight factors were similar to factors of the IPQ-R. Emotional representations included 6 items and this factor explained 19.71% of the items’ variance. Illness coherence included 5 items (explaining 7.58% of the items’ variance). Consequences of addiction included 5 items (explaining 5.47% of the items’ variance). Timeline chronic included 3 items (explaining 4.59% of the items’ variance). Personal control included 4 items (explaining 4.16% of the items’ variance). Timeline cyclical included 4 items (explaining 3.86% of the items’ variance). Treatment control included 3 items (explaining 3.55% of the items’ variance). High scores on each of these factors denote perceptions that addiction is emotionally stressful, understandable, has severe consequences, is a chronic condition, can be controlled by the patient, is a cyclic condition, and can be controlled by the treatment, respectively.

An eighth factor included seven items (explaining 9.41% of the items’ variance) that belonged to various subscales in the original IPQ-R (timeline acute/chronic, consequences, personal control and treatment control). These items reflect hopelessness and helplessness, for example: *nothing the patient does will affect his/her illness*, *there is very little that can be done to improve the illness*, and *there is nothing that can help the condition*. Therefore, we labeled this factor as a demoralization subscale. Since lower sum scores on this factor represent increased demoralization, we decided to reverse items in order to calculate sum scores for demoralization.

The Chronbach’s alpha ranged from 0.53 (Treatment Control) to 0.88 (Emotional Representations). Almost all factors were reliable for both countries (alpha ≥ 0.5), except the factor Timeline chronic (Cronbach’s alpha = 0.39) for Indonesia and Treatment control (Cronbach’s alpha = 0.46) for the Netherlands. Table 2 describes the factors of the perception scale, including the standardized factor loadings and the Cronbach’s alpha’s of each subscale.

**Table 2:** Factor Structure Perception Scale.

	Factor loading	Mean (SD)	Factor in IPQ-R
**Emotional Representations (α = .88)**			
Having this illness would make me feel anxious	.90	4.02 (0.81)	Emotional Representations
Having this illness would make me feel afraid	.89	3.99 (0.83)	Emotional Representations
Having this illness would make me feel upset	.69	3.98 (0.81)	Emotional Representations
Having this illness would make me feel depressed	.67	4.04 (0.76)	Emotional Representations
Having this illness would make me feel angry	.63	3.78 (0.88)	Emotional Representations
Having this illness would worry me	.50	4.04 (0.93)	Emotional Representations
**Demoralization (α = .81)**			
There is nothing which can help the condition (r)*	.69	4.28 (0.81)	Treatment Control
Nothing the patient does will affect his/her illness (r)*	.62	4.14 (0.81)	Personal Control
This illness will pass quickly (r)*	.57	4.06 (0.83)	Timeline Acute/Chronic
The illness will last a short time (r)*	.57	4.33 (0.75)	Timeline Acute/Chronic
The illness is easy to live with (r)*	.53	4.36 (0.88)	Consequences
There is very little that can be done to improve the illness (r)*	.52	3.87 (0.82)	Treatment Control
The actions of the patient will have no affect on the outcome of the illness (r)*	.51	4.07 (0.82)	Personal Control
**Illness Coherence (α = .86)**			
I don't understand the illness (r)	.85	3.50 (0.95)	Illness Coherence
The illness is a mystery to me (r)	.79	3.60 (1.0)	Illness Coherence
The symptoms of the condition are puzzling to me (r)	.66	3.62 (0.95)	Illness Coherence
The illness doesn’t make any sense to me (r)	.62	3.88 (0.86)	Illness Coherence
I have a clear picture or understanding of the condition	.55	3.25 (0.83)	Illness Coherence
**Consequences (α = .77)**			
The illness has serious financial consequences	.68	4.15 (0.72)	Consequences
The illness causes difficulties for those who are close to the patient	.66	4.17 (0.64)	Consequences
The illness strongly affects the way others see the patient	.57	4.20 (0.72)	Consequences
The illness has major consequences on the patients’ life	.50	4.49 (0.65)	Consequences
The illness is a serious condition	.44	4.28 (0.64)	Consequences
**Timeline Chronic (α = .67)**			
I expect the patient will have this illness for the rest of his/her life	.70	3.13 (1.0)	Timeline Acute/Chronic
The illness is likely to be permanent rather than temporary	.67	3.61 (0.94)	Timeline Acute/Chronic
The illness will last for a long time	.49	4.04 (0.66)	Timeline Acute/Chronic
**Personal Control (α = .67)**			
The course of the illness depends on the patient	.60	3.75 (0.78)	Personal Control
The patient has the power to influence the illness	.60	4.00 (0.74)	Personal Control
What the patient does can determine whether his/her illness gets better or worse	.56	3.95 (0.66)	Personal Control
There is a lot which the patient can do to control his/her symptoms	.42	3.34 (0.88)	Personal Control
**Timeline Cyclical (α = .63)**			
The symptoms come and go in cycles	.66	3.02 (0.92)	Timeline Cyclical
The illness is very unpredictable	.53	3.15 (0.93)	Timeline Cyclical
The patients go through cycles in which the illness gets better and worse	.49	3.43 (0.81)	Timeline Cyclical
The symptoms of the illness change a great deal from day to day	.47	3.36 (0.79)	Timeline Cyclical
**Treatment Control (α = .53)**			
Treatment can control the illness	.57	3.81 (0.66)	Treatment Control
Negative effects of the illness can be prevented (avoided) by treatment	.54	3.45 (0.84)	Treatment Control
Treatment will be effective in curing the illness	.44	3.19 (0.85)	Treatment Control
**Deleted item**			
The illness will improve in time			Timeline Acute/Chronic

(r)Reverse score

*We proposed not to reverse this for the IPQ-A

The maximum likelihood analysis identified four factors for illness attribution with eigenvalue greater than 1. Three items (*diet*, *bad luck*, hereditary) did not have a standardized factor loading above .40 on any factor. After deleting those items, four factors accounted for a total 58.45% of the items’ variance. Two of the four factors were similar to the factors of the IPQ-R. Psychological Attributions included 6 items (explaining 25.67% of the items’ variance). Risk Factors included 6 items and (explaining 16.85% of the items’ variance). This subscale contains three items that belonged to other subscales (immunity and accident of chance) in the original IPQ-R. The third factor, labeled Smoking/Alcohol, included two items (explaining 8.67% of the items’ variance). The last factor labeled overwork included only one item (explaining 7.26% of the items’ variance).

The Cronbach’s alpha was 0.71 for risk factors, 0.78 for smoking/alcohol, and 0.82 for psychological attribution. The reliability for the overwork subscale could not be analyzed because this factor consisted of only one item. All the factors were reliable for both countries. Table 3 describes the factors of the attribution scale, including the standardized factor loadings and the Cronbach’s alpha of each subscale.

**Table 3:** Factor Structure Attribution Scale.

	Factor loading	Mean (SD)	Factor in IPQ-R
**Psychological Attributions (α = .82)**			
Mental attitude e.g. thinking about life negatively	.82	4.26 (0.63)	Psychological attribution
Emotional state e.g. feeling down, lonely, anxious, empty	.71	4.33 (0.59)	Psychological attribution
Family problems or worries	.71	4.28 (0.60)	Psychological attribution
The patients’ own behavior	.63	4.26 (0.60)	Risk factors
Stress or worry	.51	4.22 (0.58)	Psychological attribution
Personality	.43	4.15 (0.60)	Psychological attribution
**Risk Factors (α = .71)**			
Pollution in the environment	.68	2.26 (1.02)	Immunity
Altered immunity	.65	2.63 (0.98)	Immunity
A Germ or virus	.59	1.94 (0.86)	Immunity
Poor medical care in the past	.50	2.93 (0.98)	Risk factors
Ageing	.44	2.87 (0.98)	Risk factors
Accident or injury	.41	3.20 (0.97)	Accident or chance
**Smoking-Alcohol (α = .78)**			
Smoking	.80	3.96 (0.80)	Risk factor
Alcohol use	.73	4.09 (0.72)	Risk factor
**Overwork**			
Overwork	.52	3.97 (0.68)	Psychological attribution
**Deleted items**			
Diet or eating habits			Risk factors
Chance or bad luck			Accident or chance
Hereditary—it runs in my family			Risk factors

### Correlations between the IPQ-A Subscales

Table 4 describes the correlations between subscales. Several illness perception subscales correlated, with maximum correlations for Illness Coherence with Demoralization (R = -.47, p < .01) and Consequences with Emotional Representations (R = .44, p < .01). Some attribution subscales were correlated, with the strongest correlation for the Risk Factors subscale with Smoking/Alcohol (R = .50, p < .01).

**Table 4:** Correlations between IPQ-A Subscales.

	1	2	3	4	5	6	7	8	9	10	11	12
Emotional Representations												
Demoralization	0.35**											
Illness coherence	0.21**	-0.47**										
Consequences	0.44**	-0.43**	0.23**									
Timeline chronic	0.22**	-0.21**	0.20**	0.25**								
Personal control	0.05	-0.19**	-0.09**	0.17**	0.01							
Timeline cyclical	0.11**	-0.01	-0.16**	0.08**	0.06	0.19**						
Treatment control	0.05	-0.10**	0.01	0.09**	-0.13**	0.21**	0.04					
Psychological Attributions	0.29**	-0.24**	0.02	0.34**	0.02	0.29**	0.10**	0.14**				
Risk factors	-0.12**	0.24**	-0.13**	-0.18**	-0.07**	-0.07**	0.06	0.01	-0.00			
Smoking/alcohol	0.09**	-0.04	0.01	0.12**	0.08**	0.04	0.07*	0.03	0.26**	0.50**		
Overwork	0.20**	-0.17**	0.13**	0.20**	0.07*	0.06	0.03	0.05	0.43**	0.14**	0.27**	

** p < .01 (two-tailed)

* p < .05 (two-tailed)

### Discriminant validity

First, Dutch medical students were less demoralized and more often believed that addiction is a chronic, emotionally stressful condition with severe consequences, compared to Indonesian medical students (see Table 5). They also more often thought that they understand addiction. Medical students from the Netherlands more often attributed addiction to overwork (p < .01) than their Indonesian counterparts.

**Table 5:** Perceptions of Addiction among Medical Students.

	Indonesia (n = 320)		Netherlands (n = 107)		F(1,425)	P
	mean	SD	mean	SD		
**Perception**						
Emotional Representations	3.67	0.68	3.96	0.61	15.05	< .01
Demoralization	2.19	0.55	1.76	0.35	57.25	< .01
Illness Coherence	2.98	0.56	3.73	0.50	150.57	< .01
Consequences	4.09	0.61	4.22	0.37	4.34	.04*
Timeline Chronic	3.28	0.58	3.62	0.49	30.39	< .01
Patient Control	3.92	0.58	3.69	0.49	12.87	< .01
Timeline Cyclical	3.32	0.53	3.27	0.57	0.69	.41
Treatment Control	3.56	0.62	3.46	0.48	2.05	.15
**Attribution**						
Psychological Attributions	4.27	0.55	4.29	0.36	0.05	.83
Risk Factors	2.82	0.66	2.54	0.59	15.06	< .01
Smoking/Alcohol	3.54	0.66	3.64	0.65	1.73	.19
Overwork	3.81	0.83	4.09	0.52	11.29	< .01

*significant difference (p<0.05)

Second, Dutch medical students less believed that addiction has severe consequences and is emotionally stressful, compared to psychology students (p < .01) and educational sciences students (p < .01, see Table 6). They also had more demoralized concepts of addiction (p < .01), understood addiction less well (p = .01), believed more on the cyclical timeline of addiction (p < .01), and considered less psychological factors as a cause of addiction (p = .04), compared to psychology students.

**Table 6:** Perceptions of Addiction among Students in the Netherlands.

	Medicine (n = 107)		Psychology (n = 287)		Educational Science (n = 121)		F(2,512)	P
	mean	SD	mean	SD	mean	SD		
**Perception**								
Emotional Representations	3.96	0.61	4.25	0.56	4.26	0.47	12.25	< .01^a^^,^^b^
Demoralization	1.76	0.35	1.59	0.33	1.69	0.37	10.43	< .01^a^
Illness Coherence	3.73	0.50	3.95	0.62	3.74	0.58	8.67	< .01^a^
Consequences	4.22	0.37	4.38	0.40	4.37	0.37	7.00	< .01^a^^,^^c^
Timeline Chronic	3.62	0.49	3.68	0.67	3.60	0.65	0.78	.49
Patient Control	3.69	0.49	3.65	0.53	3.62	0.54	0.65	.52
Timeline Cyclical	3.27	0.57	3.07	0.64	3.43	0.59	15.28	< .01^a^
Treatment Control	3.46	0.48	3.51	0.53	3.37	0.53	2.78	.06
**Attribution**								
Psychological Attributions	4.29	0.36	4.30	0.35	4.30	0.37	0.05	.95
Risk Factors	2.54	0.56	2.65	0.57	2.47	0.55	4.65	.01
Smoking/Alcohol	3.64	0.65	3.62	0.65	3.48	0.69	2.47	.09
Overwork	4.09	0.52	4.09	0.55	4.07	0.60	0.10	.91

^a^ significant difference between medical and psychology students (p < .01)

^b^ significant difference between medical and educational science students (p < .01)

^c^ significant difference between medical and educational science students (p < .05)

Finally, medical students had more demoralized concepts of addiction (p < .01) and believed stronger that treatment can control addiction (p = .02), compared to medical doctors (see Table 7). They also perceived less understanding of addiction (p < .01) and perceived addiction as less chronic (p < .01), with less severe consequences (p < .01) and less stressful (p = .02) than medical doctors. Medical students attributed addiction more often to psychological and risk factors (p < .01), compared to medical doctors.

**Table 7:** Perceptions of Addiction among Students and Professionals.

	Medical Students (n = 427)		Medical Doctors (n = 165)		F(1,590)	P
	mean	SD	mean	SD		
**Perception**						
Emotional Representations	3.75	0.67	3.89	0.72	5.46	.02
Demoralization	2.08	0.54	1.67	0.34	84.15	< .01
Illness Coherence	3.17	0.64	3.75	0.69	93.76	< .01
Consequences	4.13	0.56	4.27	0.42	8.53	.01*
Timeline Chronic	3.37	0.58	3.94	0.67	107.59	< .01
Personal Control	3.86	0.57	3.77	0.45	3.41	.07
Timeline Cyclical	3.31	0.54	3.36	0.58	0.71	.40
Treatment Control	3.53	0.59	3.41	0.54	5.92	.02*
**Attribution**						
Psychological Attributions	4.28	0.51	4.12	0.40	12.60	< .01
Risk Factors	2.75	0.65	2.50	0.58	19.20	< .01
Smoking/Alcohol	3.57	0.66	3.68	0.64	3.25	.07
Overwork	3.89	0.78	3.92	0.65	0.40	.53

*significant difference p < .05

## Discussion

This is the first study to measure illness perceptions on substance addiction among health professionals. Factor analysis revealed a largely similar factor structure for the IPQ-A among health professionals as the original version of the IPQ among patients, with moderate to good internal consistency. The IPQ-A differentiated perceptions on addiction between medical students from different cultural backgrounds (Indonesia and the Netherlands), students with different educational backgrounds (medicine, psychology, educational science), and participants with different educational levels (medical students and medical doctors), indicating discriminant validity.

These results indicated that the IPQ-A is a valid instrument to measure health professionals’ illness perception of addiction. Such an instrument is highly needed given the enormous variation in perceptions of addiction and its possible relationship with attitude and stigma. Since patients with addiction often perceive stigma and negative attitudes, also from health care professionals [7, 45], instruments that can help identify potential factors that may contribute to such stigma, like illness perceptions, are of great relevance. Given its acceptable psychometric properties, the IPQ-A may be such an instrument.

Our factor analysis revealed seven perception subscales, which is identical to the subscales of the original IPQ-R: timeline chronic, severe consequences, personal control, treatment control, illness coherence, timeline cyclical, and emotional representations [21]. However, one item did not load into these factors and was eliminated. In addition, we identified one new factor representing hopelessness and helplessness perception of addiction (consisting of seven items). Items loading into this factor fit with the idea that addiction is a hopeless and helpless condition that cannot be cured. This is highly relevant, since indeed healthcare professionals often belief that nothing can be done to help patients with an addiction, which reflects hopelessness and helplessness [7, 46]. On the other hand, patients with addiction often express feelings of hopelessness and helplessness, which might influence the therapeutic alliance and treatment [47–50]. We therefore propose to add this demoralization subscale to the IPQ-A for health professionals.

Our factor analysis revealed two attribution subscales (out of four) that are identical to subscales of original the IPQ-R: psychological attribution and risk factors [21]. Two subscales of the IPQ-R could not be replicated in our factor analysis: immunity and accident or chance. Items of these two IPQ-R subscales loaded into the risk factor subscale in our sample. As a result, the risk factor subscale of the IPQ-A is slightly different compared to the original IPQ-R, since four more items (diet or eating habits, ageing, chance or bad luck and accident or injury) loaded into this factor. Two other major differences between the observed factor structure of the IPQ-A and the original IPQ-R are that the items alcohol use and smoking loaded together on one new factor (and not onto the Risk Factors subscale as in the IPQ-R) and that the item overwork loaded alone onto a factor. Finally, three items (diet, bad luck, and hereditary) did not load into any of the factors and were eliminated. However, it is important to note that heritability is recognized as an important factor in the vulnerability to develop addiction, explaining about 50–60% of the risk [51].

The differences observed between the results from our factor analysis of the IPQ-A among health professionals and the original version of the IPQ-R for other conditions could be interpreted in the light of the specific relevance of alcohol and tobacco use as causal factors for the development of substance use disorders [52] [15, 53]. When asking health professionals about potential causes contributing to addiction, it makes sense that these items load to distinct factors and not together with other more general potential risk factors like chance, accidents or immune related factors.

Our exploratory factor analysis showed moderate to good internal consistency, in line with the original version of the IPQ-R [21]. In contrast, one previous study applying the IPQ-R to assess illness perceptions among health professionals on schizophrenia showed poor internal consistency in confirmatory factor analysis [33]. Similarly, poor internal consistency has been observed when applying the IPQ-R to assess illness perceptions among injecting drug users in China [32]. Therefore, we decided to apply exploratory factor analysis and not confirmatory factor analysis. By doing so, we were able to adapt the instrument for the assessment of illness perceptions on addiction, resulting in the IPQ-A, with good internal consistency for the assessment among health professionals. It may well be that psychometric evaluation of the IPQ when applied in patients with substance use disorders will indicate a different factor structure. This has particular relevance when comparing illness perceptions between patients and their health care providers.

IPQ-A subscales have moderate to good internal consistency, except for the subscale treatment control (Dutch sample: α = .46, Indonesian sample α = .73, total sample: α = .53). The treatment control subscale consists of three items: *negative effects of the illness can be prevented (avoided) by treatment*, *treatment can control the illness*, *and treatment will be effective in curing the illness*. Though these items do reflect aspects of treatment control, in the context of addiction they might represent rather different viewpoints. Where prevention of negative effects seems related to harm reduction strategies, control and cure of the illness may be more related to the recovery of the condition. Though harm reduction and cure are both related to treatment, it is not surprising that consistency over these three items is moderate. Since treatment control is both a central aspect of illness perception in Leventhal’s framework on illness perceptions and rather relevant in the context of illness perceptions on addiction among health professionals, we kept this factor in the IPQ-A, although the Cronbach’s alpha value is relatively low.

Though different independent subscales in the IPQ-A could be identified in our factor analysis, most subscales did show weak correlations with each other (correlation coefficient < .4). An association between different aspects of illness perception for a certain condition is in line with previous studies, showing for example associations between control and coherence dimensions [21]. Such associations could be expected, for example high levels on the subscale demoralization are logically associated with more negative other perceptions on addiction, e.g. uncontrollable, severe consequences and stressful.

Finally, the current study also showed significant differences in illness perception of addiction between health professionals with different 1) cultural backgrounds 2) educational backgrounds, and 3) levels of experience. These results underscore the discriminant validity of the IPQ-A, providing a tool for future studies on differences on illness perceptions of addiction between different health professionals and its relevance for example the therapeutic relationship, treatment outcome, et cetera.

The results of our study should be seen in the light of its strengths and limitations. Though we have been able to recruit a large sample of various health professionals, in different countries and with different levels of clinical experience, our results might not be generalized to other health professionals (for example psychiatrists or other medical specialists) and professionals in other countries. Since all medical specialists, especially psychiatrists, meet patients with addiction in their clinical practice, and medical specialists may have distinct illness perceptions on addiction compared to medical students or addiction specialists in training, future studies should examine whether our findings also hold for these professionals. Second, this study assessed participants’ perceptions cross-sectionnally. Therefore retest reliability could not be evaluated. Third, it is important to explore the factor structure of the IPQ-R among patients with addiction. For future comparisons of illness perceptions of addiction between patients and health professionals it is important to create comparable questionnaires, with identical subscale structure. Finally, it cannot be fully ruled out that minor changes applied to some items of the original IPQ-R in order to make them appropriate for health care professionals, might affected the meaning of these items. Since these are only minor changes applied to four items major effects on the psychometric properties of the IPQ-A are unlikely.

Despite these limitations, this study showed good psychometric properties of the IPQ-A in health care professionals. Therefore, the IPQ-A can be considered a valid and reliable tool to measure healthcare professionals’ perceptions of substance addiction. This instrument can be used in future studies on perceptions of addiction among health care professionals. Such studies should explore healthcare professionals’ perceptions of addiction in relation with their attitudes towards patients with addiction, and the effect on the therapeutic relationship and treatment outcome. Furthermore, the IPQ-A may have relevance to evaluate the development of health professionals’ perceptions of addiction during professional education and training. As such, the IPQ-A may be a useful tool for reflection techniques in training programs and the evaluation of effect of training programs.
